# SNP rs10748643 determines CD39 expression in T and NK cells through altering NFIC binding affinity rather than interfering with RNA splicing

**DOI:** 10.3389/fimmu.2025.1679064

**Published:** 2025-12-01

**Authors:** Chenxin Gan, Qian Wu, Jorg J. Goronzy, Yongchun Zhang, Lingjie Li, Huiming Sheng, Fengqin Fang

**Affiliations:** 1 Department of Laboratory Medicine, Tongren Hospital, Shanghai Jiao Tong University School of Medicine, Shanghai, China; 2 Department of Immunology, Mayo Clinic College of Medicine and Science, Rochester, MN, United States; 3 State Key Laboratory of Microbial Metabolism and Joint International Research Laboratory of Metabolic and Developmental Sciences, School of Life Sciences and Biotechnology, Shanghai Jiao Tong University, Shanghai, China; 4 Department of Histoembryology, Genetics and Developmental Biology, Shanghai Key Laboratory of Reproductive Medicine, Key Laboratory of Cell Differentiation and Apoptosis of Chinese Ministry of Education, Shanghai Jiao Tong University School of Medicine, Shanghai, China

**Keywords:** SNP rs10748643, CD39 expression, T lymphocytes, NFIC, transcriptional regulation, RNA splicing

## Abstract

NTPDase-1 CD39, a dominant component of purinergic signaling, has proved to be a critical regulator of tissue microenvironment and cellular function particularly for T cells. We repeatedly use single-nucleotide polymorphism (SNP) rs10748643 genotype as a tag of CD39 expression in T cells in many studies. However, no study that profiles the impact of the SNP mutation on CD39 expression in all immune cells has been done yet. In this study, we used flow cytometry to measure CD39 expression in six types of immune cells across three genotypes. We revealed that SNP rs10748643 mainly determines CD39 expression in T and natural killer (NK) cells. In contrast, it has the least influence on monocytes, B cells, and granulocytes. These immune cells demonstrate constitutive CD39 expression, which is different from T and NK cells. We applied bioinformatic tools and bench experiments to disclose the underlying mechanism. We found that the amount of each variant is barely affected by SNP mutation in B cells, whereas almost all variants are significantly impacted by SNP mutation in T cells. Such discrepancy in T and B cells does not support the hypothesis that SNP rs10748643 interferes with the splicing efficiency of CD39 mRNA. MicroRNA regulation was also excluded. Transcriptional factor regulation was emphasized because conserved motif sequence of NFIC exactly covers the SNP site. Using Cleavage Under Targets and Tagmentation (CUT&Tag) technology, we found that the binding affinity of NFIC decreases when SNP mutates from A to G, causing the reduced inhibition of NFIC on CD39 expression in T cells. The NFIC inducer may be another potential approach to restrain CD39 expression for improving the immunosuppressive microenvironment.

## Introduction

Purinergic signaling and its associated molecules widely exist in various immune cells and vascular endothelial cells (1). Adenosine triphosphate (ATP), released from activated cells or apoptotic cells, is hydrolyzed by ATPase CD39 (also ADPase) into adenosine monophosphate (AMP), which further breaks down to adenosine via AMPase CD73. In the presence of these enzymes, inflammatory environments dominated by ATP and adenosine diphosphate (ADP) are converted to anti-inflammatory milieu filled with AMP and adenosine. Theoretically, CD39 is more powerful in modifying the microenvironment than other enzymes because it not only breaks down ATP and ADP, but also produces AMP and adenosine. However, AMPase CD73 has a sole role in generating adenosine. From this perspective, CD39 is a dual-functional molecule whereas CD73 is a mono-functional molecule. Thus, targeting CD39 is considered to be a more efficient way to reverse the inhibitory local environment.

A bunch of transcription factors (TFs) were experimentally confirmed to regulate CD39 transcription especially in T cells, including FOXP3, SP1, RUNX3, BCL6, CREB, and GATA3 (2–6). More interestingly, Robson’s group first discovered that single-nucleotide polymorphism (SNP) at rs10748643 significantly modulates CD39 expression in the lymphoblastoid cell line (LCL) (7). The follow-up studies (3, 8) repeatedly confirmed this phenomenon in T lymphocytes. T cells with AA alleles barely express CD39, while T cells with GG alleles highly express CD39 and heterozygote stays in the middle. The stimulation of T cells with anti-CD3/CD28 antibodies could not change this pattern, but even widen the difference between genotypes (8).

There are plenty of lines of evidence (3, 8, 9) showing that purinergic signaling plays a pivotal role in fine-tuning T-cell function and differentiation. T lymphocytes with AA alleles that express little CD39 are prone to differentiate into follicular helper T cells (3) or long-term memory T cells (8). In contrast, T cells with GG alleles that express a high level of CD39 are easily differentiated into Th1 cells with robust effector function (3). It is worth noting that CD39 upregulation on regulatory T cells (Treg) was considered to be a prerequisite of responsiveness of methotrexate therapy in patients with rheumatoid arthritis (10). From all the data we obtained from the benchwork, we found that it was very difficult to induce CD39 expression in T cells with the AA genotype at rs10748643, no matter which stimulus was used and how strong the stimulus signal was. CD39 is an additional biomarker for regulatory T cells, but it is not necessary. CD39 expression on human Treg cells is also rigorously regulated by SNP rs10748643. Thus, we have enough reason to speculate that these patients with no responsiveness were AA genotype at rs10748643, although the authors did not realize that. The immunosuppressive role of CD39 and its application in autoimmune diseases were gradually appreciated. It is worth mentioning that the proportion of AA genotype varies between 16% and 66% with the least in Caucasian people and the most in Chinese people (see the data below). No matter in which population and which genotype, the subpopulation is not small. If a specific genotype is confirmed to be associated with the incidence of a certain disease, the responsiveness of a specific treatment, or a specific function of immune cell, the clinical significance could not be neglected. The pioneering work by Robson’s group disclosed that SNP rs10748643 influenced human susceptibility to Crohn’s disease. The AA genotype was significantly enriched in the patients with colitis (7).

Nevertheless, profiling the extensive influence of SNP rs10748643 on CD39 expression in different immune cells is the first thing to do. To our knowledge, our study investigates for the first time how SNP mutation regulates CD39 expression in six types of immune cells. The underlying mechanism was particularly dissected. We found that SNP rs10748643 mainly affected CD39 transcription in T lymphocytes and natural killer (NK) cells with an inducible CD39 expression. Instead of interfering with RNA splicing, we disclosed that SNP mutation from A to G loosens the binding of TF NFIC to the DNA sequence, resulting in the reduced inhibition on CD39 transcription in T cells.

## Results

### The distribution of SNP rs10748643-associated genotypes in a Chinese population

As we mentioned before, SNP rs10748643-associated AA, AG, and GG genotypes are all very common in the worldwide population. However, the proportion of each genotype varies among people from different human origins. People of Caucasian or European or African ancestry have almost equal frequency of the A or G allele at rs10748643 (**Table 1**). In contrast, the A allele prevails most in Chinese people, with the frequency up to 80% (**Table 1**). AA and AG alleles dominate in Chinese people, accounting for two-thirds and one-thirds, respectively (**Table 2**). GG alleles only take up 5% in Chinese people. Such discrepancy of genotype distribution among different ancestries may cause each population to be susceptible to different diseases.

**Table 1:** Allele frequency of SNP rs10748643 in four populations with different ancestries.

Population	EUR*	AFR*	JPT*	CHB
A	0.456	0.458	0.637	0.780
G	0.544	0.542	0.363	0.220

*Data are acquired from NCBI dbSNP (https://www.ncbi.nlm.nih.gov/snp/rs10748643). EUR, AFR, JPT, and CHB represent the population with European, African, Japanese, and Chinese ancestry, respectively. According to the dbSNP website, EUR and AFR data are computed from dbGaP studies and JPT data are computed from the literature (11).

**Table 2:** SNP rs10748643 genotyping result of 255 Chinese healthy donors.

Genotype	AA	AG	GG	Total
People (no)	157	84	14	255
Percentage (%)	61.57	32.94	5.49	100

### Profiling SNP rs10748643 regulation on CD39 expression in different immune cells

**Figure 1:**
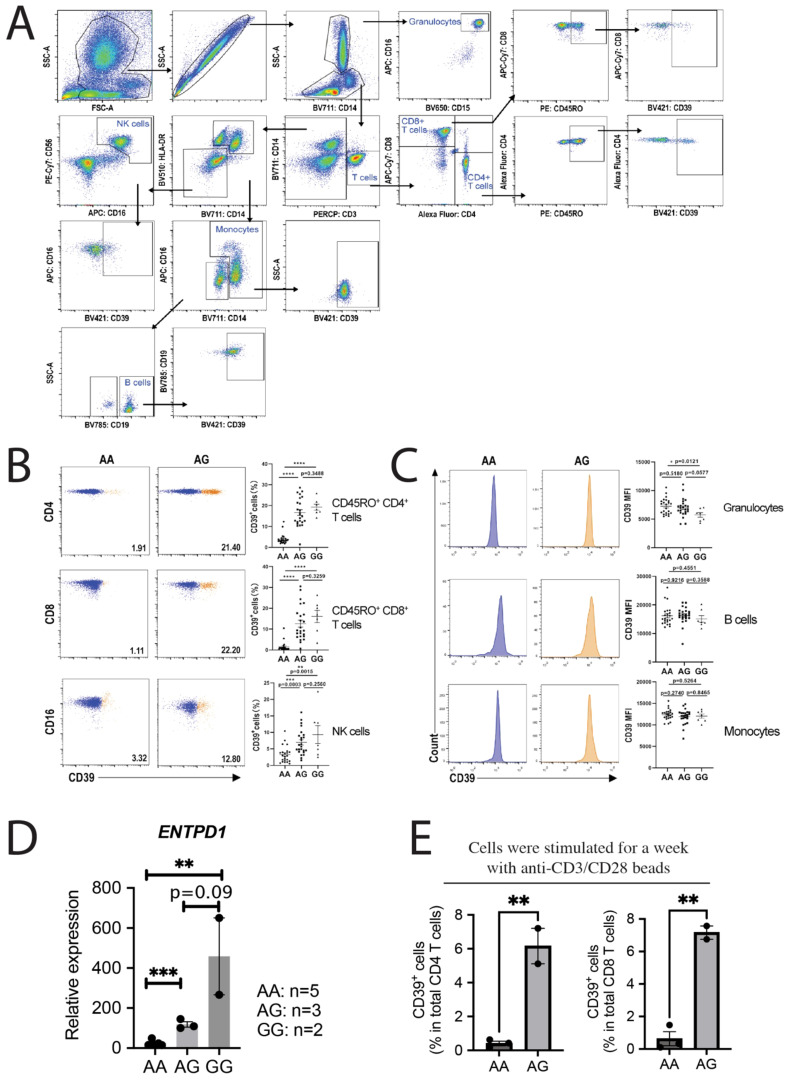
SNP rs10748643 determines CD39 transcription mostly in T and NK cells with an inducible CD39 expression. **(A)** The staining and gating strategy of human peripheral immune cells. **(B)** The frequency of CD39^+^ cells in memory CD4 and CD8 T and NK cells with AA, AG, or GG alleles. **(C)** CD39 expression level quantified by flow cytometry with median fluorescence intensity (MFI) in granulocytes, monocytes, and B cells with AA, AG, or GG alleles. **(B, C)** Left shows representative data of flow cytometry in the AA and AG group. Right shows the summary expressed as mean ± SEM. The number of samples in the AA, AG, and GG group is 23, 24, and 7, respectively. **(D)** The differential expression of *ENTPD1* mRNA in resting T cells with different genotypes quantified by real-time qPCR. **(E)** Purified T cells were stimulated for a week using anti-CD3/CD28 Dynabeads before detecting the frequency of CD39^+^ cells by flow cytometry. *p*-values were obtained by two-tailed unpaired *t*-test. *p< 0.05, **p< 0.01, ***p< 0.001, ****p< 0.0001.

After knowing the above general information, we urge to observe the regulation of SNP rs10748643 on CD39 expression in six types of immune cells, namely, granulocytes, monocytes, B lymphocytes, NK cells, and memory CD4 and CD8 T cells as control. We collected peripheral blood from healthy donors. After lysis of red blood cells, all leukocytes were stained with antibody cocktail targeting CD3/CD4/CD8/CD45RO/CD14/CD16/CD56/CD15/HLA-DR/CD19/CD39 to separate the abovementioned immune cells according to the strategy provided by Boesch et al. (12) (**Figure 1A**). We found that granulocytes, monocytes, and B cells demonstrate constitutive CD39 expression. Conversely, T lymphocytes and NK cells show the inducible expression pattern. Most importantly, SNP rs10748643 seems to only regulate CD39 expression on T and NK cells (**Figure 1B**), not on granulocytes, monocytes, and B cells (**Figure 1C**). The differential CD39 expression between AA and AG genotypes was observed and confirmed in T and NK cells (**Figure 1B**). We also saw the trend of CD39 upregulation from the AG to GG group, and statistical insignificance may be due to the insufficient number of donors in the GG group (**Figure 1B**). In addition, we need to emphasize that the differential expression of CD39 protein between AA, AG, and GG genotypes was reproduced by real-time quantitative polymerase chain reaction (qPCR) detecting *ENTPD1* mRNA level across three genotypes (**Figure 1D**). Such consistency between protein and mRNA level excludes the involvement of translational and post-translational regulation by SNP rs10748643. As we expected, human T cells with the AA genotype hardly express any CD39, whether the cells are resting or activated (**Figure 1E**).

### Immune cells with constitutive CD39 expression show the open and accessible chromatin in the *ENTPD1* gene region compared to the cells with inducible CD39 expression

**Figure 2:**
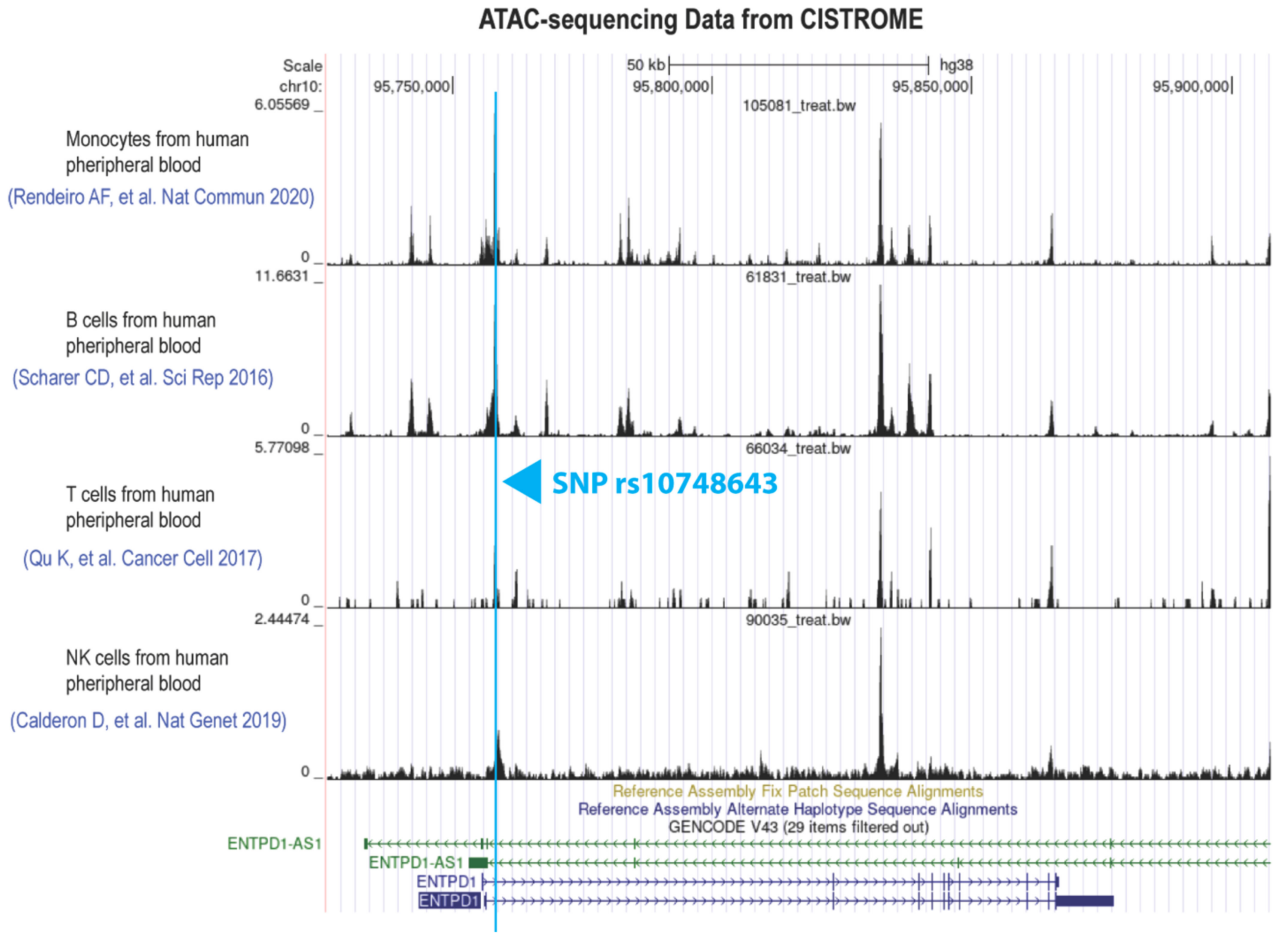
Monocytes and B cells with constitutive CD39 expression have much more chromatin openness in the *ENTPD1* gene region compared to T and NK cells. These ATAC-seq data were downloaded from the CISTROME database.

After knowing the CD39 expression pattern in different immune cells, we wondered why some immune cells (granulocytes, monocytes, and B cells) have constitutive CD39 expression but T and NK cells have inducible CD39 expression. Thanks to the CISTROME database, we found the huge difference in chromatin openness in the *ENTPD1* gene region between these two types of cells. ATAC-sequencing data showed that monocytes and B cells have rather open and accessible chromatin at the *ENTPD1* gene region for transcriptional regulation (**Figure 2**). Conversely, that region is quite closed in resting T and NK cells (**Figure 2**).

### SNP rs10748643 does not have equal regulation on CD39 variants in T and B cells; the observation does not support the hypothesis that SNP mutation interferes with RNA splicing efficiency

**Figure 3:**
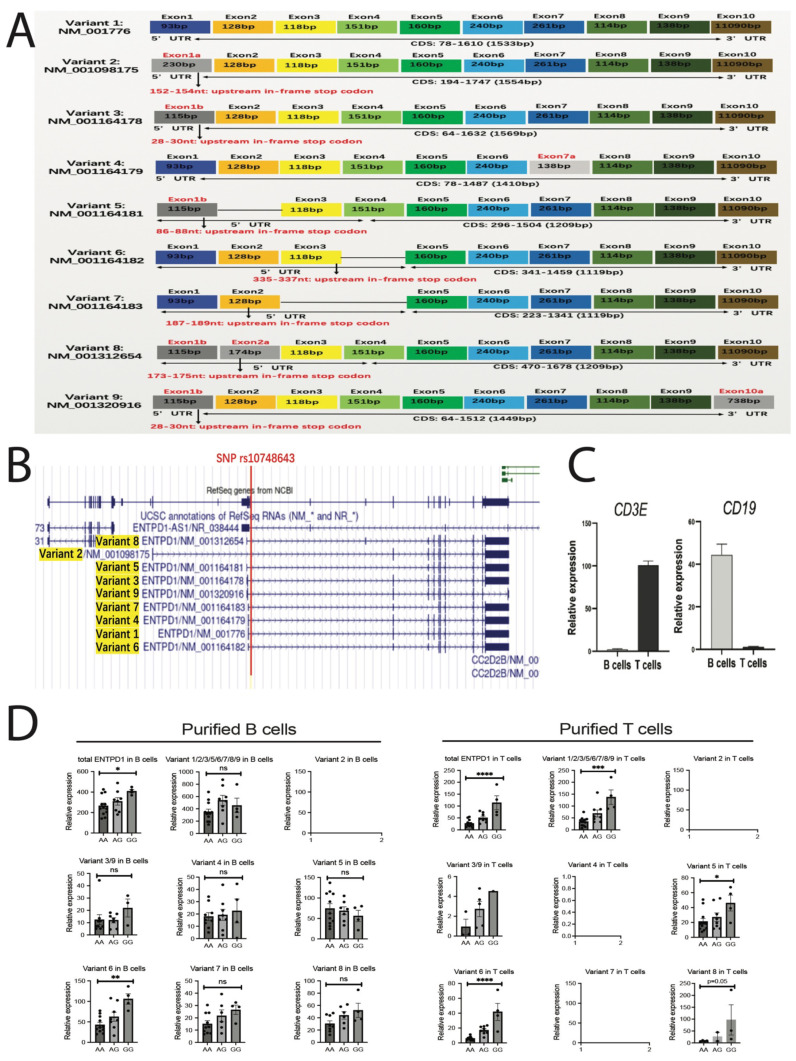
SNP rs10748643 significantly regulates almost all variants of CD39 mRNA in T cells but not in B cells; such inconsistency in T and B cells do not support the hypothesis that RNA splicing efficiency is altered by SNP mutation. **(A)** The schematic figure shows the gene structures of 9 *ENTPD1* mRNA variants. Among them, 7 variants (except variants 1 and 4) contain upstream in-frame stop codon in the 5′UTR region. **(B)** The schematic figure from the genome browser shows that SNP rs10748643 is very close to exon 1 (approximately 500–1,000 bp distance from exon 1). The generation of each variant needs to get rid of the intron containing SNP rs10748643. The location of SNP did not show the preference of splicing regulation on some specific variants. **(C)** Confirming the purity of isolated T and B cells by quantifying *CD3E* and *CD19* gene expression in both cell types. The data were shown as $2^{-\Delta \Delta Ct}$ and normalized to the lowest one in each figure. **(D)** Quantifying each variant of CD39 mRNA stratified by genotype in purified T and B cells. One-way ANOVA was applied for comparing three groups. ns (not significant): p>0.05, *p< 0.05, **p< 0.01, ***p< 0.001, ****p< 0.0001.

Now, the key question is why this particular single point mutation could significantly control CD39 expression in T and NK cells. Three possible mechanisms were considered here. One mechanism refers to RNA splicing. SNP rs10748643 is located in the first intron of the *ENTPD1* gene, with an approximately 500–1,000 bp distance from the first exon and also very close to the promoter sequence (**Figure 3B**). According to the structure of the spliceosome, which is the most common mechanism to remove all introns in pre-mRNAs, there are three crucial splicing elements in intron: a 5′ donor site with invariant sequence GU, a 3′ acceptor site with invariant AG sequence, and the branch site near the 3′ end of the intron, which includes polypyrimidine tract and upstream branchpoint containing an adenine nucleotide involved in lariat formation (13). Any point mutations in these critical regions, especially the sequence from branchpoint to the 3′ end of the intron, have a great chance to interfere with RNA splicing. However, the location of SNP rs10748643 is quite close to the 5′ end of the first intron (**Figure 3B**) and perfectly avoids the regions that are crucial to form the lariat splicing structure. Thus, the chance of this particular SNP interfering with RNA splicing is very small.

More importantly, we applied an array of real-time qPCR assays to support this inference. RNA splicing in different sites produces mRNA variants. Based on the NCBI reference sequences (https://www.ncbi.nlm.nih.gov/gene/953), the *ENTPD1* gene transcribes nine mRNA variants named variant 1 to variant 9. The gene structures of these variants are clearly shown in **Figure 3A**. In general, the 3′ end of all variants are quite conserved, and the difference mostly exists in the 5′ end. The sequences of variants 3 and 9 are almost identical except for exon 10. When we carefully check up on these variants, we found that the special feature of “upstream in-frame stop codon” is very common in *ENTPD1* mRNA variants. Out of nine variants, seven (except variants 1 and 4) have this nonsense codon in the 5′UTR region. Moreover, near-cognate start codons (non-AUG start codons) are very common but normally weak in the 5′UTR region. The near-cognate start codon and in-frame stop codon form upstream ORF (uORF) in frame. However, most of them are ignored and bypassed by the ribosome due to the weak start codon, or even though uORF is translated earlier, the ribosome still efficiently re-initiates the translation of the main CDS. In these cases, variants are capable of producing functional proteins. However, in some specific situations (for example, start codon in 5′UTR is very strong, or cells undergo stress), ribosome initiates uORF translation but fails main CDS translation; it will incur the premature translation termination and triggering of nonsense-mediated decay (NMD) based on the EJC (Exon–Junction complex) theory (14, 15). mRNA variants that fail to translate the main CDS could trigger mRNA degradation. Thus, we speculate that CD39 variants with upstream in-frame stop codon have a chance to incur premature translation termination and trigger NMD especially when start codon in 5′UTR is very strong, or cells undergo certain stress.

We downloaded and compared these sequences to pick up the variant-specific primers as follows: variant 2-specific, variant 3/9-specific, variant 4-specific, variant 5-specific, variant 6-specific, variant 7-specific, and variant 8-specific primers as well as a pair of primers to detect variants 1, 2, 3, 5, 6, 7, 8, and 9 simultaneously (we give it a name: variant full spectrum primers) (see **Supplementary Table 1**). There are no variant 1-specific primers due to the lack of unique sequences in variant 1 (see **Figure 3A**). However, we can calculate the relative expression of variant 1 from the total quantity deducting to the other variants. To figure out the regulation of SNP mutation on each variant, we quantified the variant-specific CD39 mRNA level in the purified B and T cells stratified by genotype. Variant 2 is not detectable in purified B cells. Variant 2, variant 4, and variant 7 are not detectable in purified T cells. However, variant 1, variant 5, variant 6, and variant 8 are the most abundant CD39 variants in both T and B cells according to the relative expression level. Most importantly, except for variant 6, all other variants are not influenced by SNP mutation in B cells. In contrast, almost all variants are significantly impacted by SNP mutation in T cells (**Figures 3C, D**). If SNP rs10748643 interferes with the RNA splicing efficiency of each variant or some variants, it should have equal influence on T and B cells, but apparently it does not. Taken together, the lines of evidence do not support the hypothesis that SNP rs10748643 interferes with the splicing efficiency of CD39 mRNA.

Another possible mechanism is SNP-mediated microRNA regulation on CD39 expression, which could be excluded by the following analysis: MicroRNAs normally have a two- to eight-nucleotide seed sequence, which mostly recognizes the 3′UTR region of mature mRNA. MicroRNA regulation normally happens in the cytoplasm after mature mRNA is produced. SNP rs10748643 is located in the first intron of the *ENTPD1* gene region, and the area should be removed during mRNA maturation in the nucleus. Thus, mature *ENTPD1* mRNAs no longer have this specific SNP, Cytoplasmic microRNAs have no chance to bind to this SNP site.

### The SNP mutation from A to G decreased the binding affinity of transcription factor NFIC to loosen its inhibition on CD39 expression in T cells

**Figure 4:**
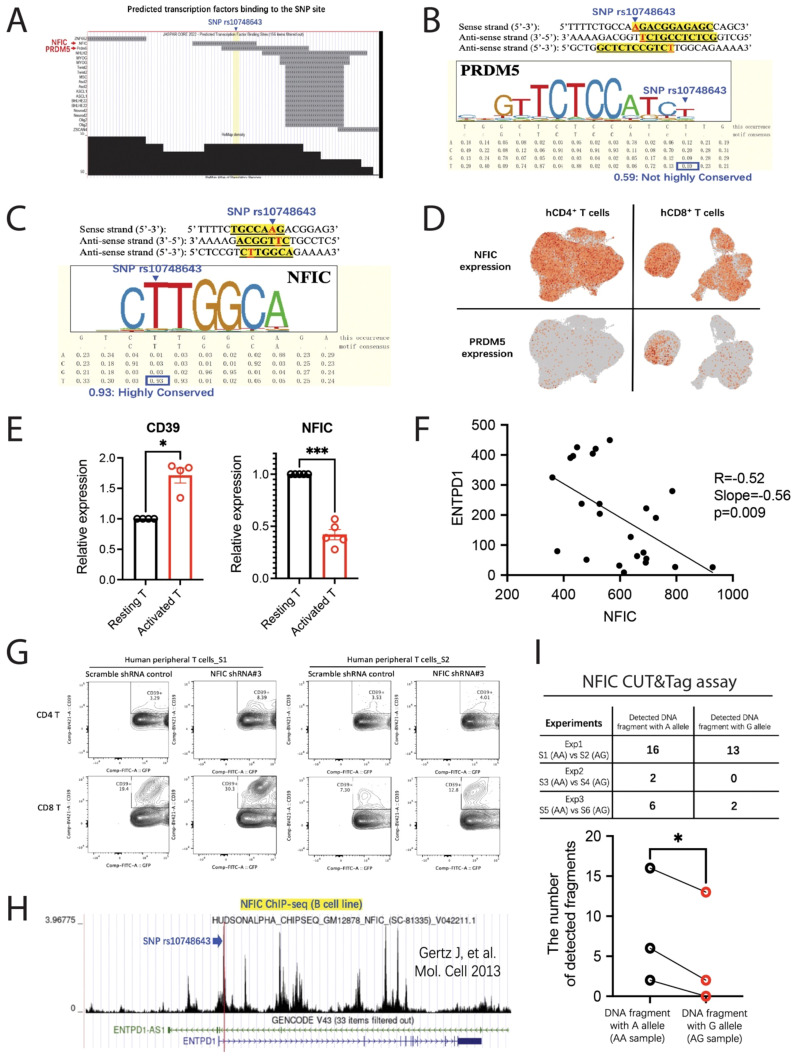
SNP rs10748643 regulates CD39 expression in T cells through altering the binding affinity of NFIC to the SNP site. **(A)** Searching for potential TF motifs in DNA sequences around the SNP site using the UCSC genome browser. **(B, C)** The demonstration of PRDM5 **(B)** and NFIC **(C)** motif consensus covering the SNP site. **(D)** The expression level of NFIC and PRDM5 in our scRNA-seq of human T cells from peripheral blood. **(E)** T-cell activation by anti-CD3/CD28 Dynabeads induced CD39 expression but inhibited NFIC transcription. **(F)** The negative correlation between NFIC and ENTPD1 mRNA level in isolated T and B cells from human peripheral blood. **(G)** NFIC knockdown using a lentivirus system with NFIC shRNA upregulated CD39 expression in primary T cells. **(H)** NFIC ChIP-seq data of a B-cell line (GM12878) downloaded from the CISTROME database. **(I)** The result of the CUT&Tag assay comparing the binding affinity of NFIC to the SNP site with A allele or G allele. Human peripheral T cells were activated for 4 days before doing the CUT&Tag assay. The experiments were repeated three times using six samples in total. Paired *t*-test was used in **(E)**, I *p< 0.05, ***p< 0.001. In addition, the statistics of simple linear regression was used in **(F)**.

The other mechanism, which is interrupted transcriptional regulation, could be possible. The DNA region around SNP rs10748643 is located in the chromatin open area, which is accessible for transcriptional regulation (see **Figure 2**). This fact supports the hypothesis that SNP rs10748643 regulates CD39 expression in T cells probably by interfering with TF binding affinity to DNA sequence. We selected the genomic region of 50-bp length flanking the SNP site and searched the potential TF motifs in it using the UCSC genome browser, which displays tracks showing the genome-wide predicted TF binding profiles based on the motif database from the JASPAR CORE collection. The motifs of TF NFIC (nuclear factor I C) and PRDM5 were found to cover the SNP site in the submitted sequence, suggesting the potential binding sites and their regulation on *ENTPD1* gene transcription (**Figure 4A**). TF NFIC has a conserved binding motif 5′C**T**TGGCA3′ (or 5′TGCCA**A**G3′). The highlighted bold letter is the exact SNP site and highly conserved in this motif (**Figure 4C**). Conversely, the motif sequence of PRDM5 is quite long and the nucleotide at the SNP site is not highly conserved in this motif (**Figure 4B**). More than this, PRDM5 is rarely expressed in human resting T cells (**Figure 4D**). Based on these reasons, PRDM5 was excluded from our candidate molecules. In contrast, NFIC expression is quite robust in human peripheral CD4 and CD8 T cells (**Figure 4D**). When we look closer at NFIC motif consensus, the A allele at rs10748643 is the preferential binding element. When A mutates to G at the SNP site, the binding affinity of NFIC is speculated to drop down a lot. However, we all know that the A allele at rs10748643 prevents CD39 transcription and the G allele promotes CD39 expression. Therefore, we are asking whether NFIC suppresses rather than promotes CD39 transcription through binding to DNA sequence with high affinity. Indeed, we found the lines of evidence as follows. We isolated human T cells from peripheral blood to culture *in vitro*. The stimulation of T cells with anti-CD3/CD28 Dynabeads increased CD39 transcription but decreased NFIC expression (**Figure 4E**). The opposite trends of these two genes before and after stimulation indicate the inhibitory regulation of NFIC on CD39 expression. Consistently, we found the solid negative correlation between CD39 and NFIC expression in isolated T and B cells (**Figure 4F**). The correlation was statistically significant. Taken together, NFIC probably inhibits CD39 expression as we expected. For further confirmation, we transferred NFIC shRNA into the activated primary T cells using the lentivirus system. The data showed that NFIC knockdown upregulated CD39 expression in human T cells (**Figure 4G**). To prove the direct binding of NFIC to the *ENTPD1* gene region especially at the SNP site, we obtained NFIC ChIP-seq data of the B-cell line from the CISTROME database. The data show that NFIC actively binds to the multiple sites in the *ENTPD1* gene region and regulates CD39 expression (**Figure 4H**). If we observe carefully, there is one marked peak exactly located at the SNP site, indicating that NFIC binds to the SNP site. The other confirmative experiment is the Cleavage Under Targets and Tagmentation (CUT&Tag) assay using anti-NFIC primary antibody. Since GG alleles at rs10748643 are quite rare in Chinese people, we collected T-cell samples with AA or AG alleles, then compare the efficiency of NFIC binding to the DNA fragments with different alleles. The data showed us that NFIC could bind to the DNA sequence either with the A allele or with the G allele. However, the binding affinity drops significantly when the nucleotide at the SNP site mutates from A to G (**Figure 4I**). The experiment was repeated three times. These findings tell us that SNP rs10748643 regulates CD39 expression in T and NK cells at least partially through changing NFIC binding affinity to the SNP site.

In summary, SNP rs10748643 controls CD39 expression in T and NK cells probably through interfering with NFIC binding affinity. The inhibitory function of NFIC on CD39 expression was observed and shows potential for clinical applications. The mechanisms referring to RNA splicing efficiency and microRNA regulation were both excluded using bench experiments and theoretical analysis.

## Discussion

Ectonucleotidase CD39 as a core molecule in purinergic signaling is mainly expressed in various immune cells and vascular endothelial cells to regulate immune response and thrombosis, respectively. We are interested in how SNP rs10748643 regulates CD39 expression in all immune cells from human peripheral blood, including granulocytes, monocytes, NK cells, B lymphocytes, and memory CD4 and CD8 T cells. Naïve T cells do not express CD39 and were excluded here. NKT and dendritic cells were not analyzed. However, the immune cells we studied here are enough to demonstrate two types of CD39 expression pattern: one is a constitutive CD39 expression and the other one is an inducible CD39 expression. We found that SNP mutation at rs10748643 mainly affects CD39 transcription in T lymphocytes and NK cells, which show an inducible expression pattern, but barely disturb CD39 expression in the cells with constitutive expression. It is worth noting that CD39 mRNA level is consistent with the protein level in T cells with AA, AG, or GG alleles to exclude the translational and post-translational regulation by SNP mutation.

The mechanism of SNP rs10748643 regulating CD39 expression is very likely attributed to the altered binding affinity of NFIC to the SNP site rather than interfering with RNA splicing efficiency. Robson’s group applied the HapMap database and the corresponding 270 LCLs to discover SNP rs10748643 as a tag for CD39 expression (7). EBV-transformed LCLs are B-cell lines made from primary B lymphocytes. The data are conflicting with ours. The reason probably hides in the dramatic differences beyond our expectation between primary B lymphocytes and B-cell lines. Several groups applied genome-wide approaches or microarray to profile primary B cells, LCLs, and T cells from the same donors with or without drug treatment. Unexpectedly, samples were largely clustered by cell types, not by individuals or drug treatment, indicating the distinct gene expression between primary B cells and LCLs transformed by the EBV virus (16, 17). They found that more than half of the genes were differentially expressed between EBV-transformed LCLs and primary B cells, even though most of these changes were of small magnitude (16). Moreover, it was reported that only 9.8% of expression quantitative trait loci (eQTL) identified in LCLs were recapitulated in primary B cells (18). SNP rs10748643 regulation on CD39 expression is not captured in primary B cells but impressively identified in primary T cells and NK cells.

The difference in methodological sensitivity may also generate different outcomes. We applied flow cytometry to detect CD39 protein level instead of using real-time Q-PCR to detect mRNA quantity, which is more sensitive. We did not see the differential CD39 expression on B cells under different genotypes, but we presume that TF NFIC also binds to the *ENTPD1* gene region in B cells to suppress CD39 expression. However, the extensive and broader chromatin openness of the *ENTPD1* gene region in B cells allows numerous TF binding and regulation so that one single point mutation is no longer important.

Taken together, SNP rs10748643 A-to-G mutation is enough to modulate CD39 expression in T and NK cells. T cells with AA alleles barely express any CD39. This phenotype could mimic the conditional knockout of the *ENTPD1* gene in T and NK cells, which is a perfect tool to study how ATPase CD39 regulates T-cell function and differentiation both *in vitro* and *in vivo*.

NFIC is a member of NFI family TFs, also including NFIA, NFIB, and NFIX. Studies researching NFIC in T cells are very few. A couple of studies refer to the role of NFIC in causing neural tube defects (19), in monocyte differentiation and leukemia cell survival (20), and in osteogenesis and bone homeostasis (21). In our study, we found that NFIC was highly expressed in resting T cells and much less in activated T cells. NFIC binds to *ENTPD1* gene loci to negatively regulate CD39 expression. SNP mutation at rs10748643 regulates CD39 transcription at least partially by altering the binding affinity of NFIC to DNA sequence.

CD39^+^ T cells were actively investigated in the field of chronic virus infection and tumor environment. CD39 is taken as an exhaustion marker (22–24). People think that constant antigen stimulation upregulates CD39. Thus, CD39^+^ T cells in tumor tissue are regarded as tumor antigen-specific T cells while CD39^−^ T cells are considered as bystander T cells (24). However, it is still unclear whether the ATPase enzyme activity of CD39 directly promotes T-cell exhaustion. Primary B cells often co-express CD39 and CD73, producing much more AMP and adenosine compared to T cells (25). Thus, CD39^high^ B cells were classified as regulatory B cells (26). On the other hand, CD39^+^ NK cells are much less studied so far. One group found that CD39^+^ NK cells also highly express CD38, which degrades NAD+ into ADPR, then generate adenosine in tandem with CD203a and CD73. CD38^+^CD39^+^NK cells were proven to have an inhibitory function on effector T cells (27). Briefly, purinergic signaling centered on ATPase CD39 plays a crucial role in regulating immune cell function and other features. CD39 and its regulators will continue to be actively investigated in the field of autoimmune disease and tumor therapy.

## Materials and methods

### Study population and cell purification

A total of 255 de-identified whole blood samples from healthy donors were leftovers after routine blood tests for physical examination. The study was ethically approved by Shanghai Jiao Tong University School of Medicine and Tongren Hospital Review Boards. Untouched total T and B cells were purified using the following commercial kits: RosetteSep Human T-cell enrichment cocktail (Cat#: 15021, STEMCELL Technologies) and RosetteSep Human B-cell enrichment cocktail (Cat#: 15024, STEMCELL Technologies). The purity of isolated cells was >95%.

### DNA isolation and SNP genotyping assays

Isolate genomic DNA from whole blood using the DNeasy Blood & Tissue Kit (Cat#: 69506, Qiagen). The procedure of identifying genotype at rs10748643 was described in detail in our previous publication (8). In brief, prepare a master mix including 10 ng of sample DNA, TaqMan Universal PCR Master Mix (Cat#: 4304437, Applied Biosystems Inc), TaqMan SNP Genotyping Assay (Cat#: 4351379, Applied Biosystems Inc, Assay ID: C_27420559_10), and Nuclease-free water for qPCR according to the manufacturer’s recommendations. The probe fluorescence signal was detected with QuantStudio 5 Real-Time PCR Systems (Applied Biosystems Inc).

### Spectral flow cytometry analysis

Whole blood samples were lysed to remove red blood cells before adding the antibody cocktail for staining. Antibodies targeting CD3/CD4/CD8/CD45RO/CD14/CD16/CD56/CD15/HLA-DR/CD19/CD39 are shown in **Supplementary Table 2**. Data analysis was performed with spectral flow cytometry (Cytek Aurora System), which allowed us to avoid the compensation step and use much more channels simultaneously compared to the DB flow cytometry system. The peripheral blood immune cell separation and gating strategy is depicted in **Figure 1** according to the literature (12).

### RNA extraction and RT-qPCR

Total RNA was extracted from purified peripheral T or B cells using the RNeasy Plus Mini Kit (Cat#: 74136, Qiagen) according to the manufacturer’s instructions. cDNA templates were synthesized using the HiScript III RT SuperMix for qPCR (+gDNA wiper) (Cat#: R323-01, Vazyme Biotech). Real-time qPCR assays were performed with the ChamQ Universal SYBR qPCR master mix (Cat#: Q711-03, Vazyme Biotech) and the Bio-Rad T100 thermal cycler (Bio-Rad). The relative expression level of the indicated genes was normalized to *ACTB*. All primer sequences used in this study are listed in **Supplementary Table 1**.

### Bioinformatic analysis

ATAC-seq data of human peripheral blood T cells, NK cells, monocytes, and B cells were downloaded from the CISTROME database (http://cistrome.org/db/#/) to compare the chromatin openness at the *ENTPD1* gene region between these cells. The UCSC genome browser (https://genome.ucsc.edu) was used to predict the potential TFs binding to the SNP site. JASPAR CORE was taken as a TF motif bank for this prediction (http://jaspar.genereg.net). NFIC ChIP-Seq data were also downloaded from the CISTROME database to confirm the direct binding of NFIC to the *ENTPD1* gene region particularly at the SNP site.

### Lentiviral transduction

NFIC shRNA and lentivirus vector containing NFIC shRNA were designed and made by HanBio company. The sequence of NFIC shRNA is as follows: 5′ GATCC GCAGAGATGGACAAGTCACCATTCAA TTCAAGAGA TTGAATGGTGACTTGTCCATCTCTG TTTTTTG 3′. The protocol of lentiviral transduction was briefly described here. First, human T cells were isolated from fresh peripheral blood, then activated with anti-CD3/CD28 Dynabeads and transduced by lentivirus with NFIC shRNA or control shRNA (10^7^ PFU per test) in the presence of 8 μg/mL polybrene (Millipore Sigma) and 10 U/mL human IL-2 (Peprotech). Cells were cultured for 4 days before doing flow cytometry. Analysis was done on lentivirus-infected GFP^+^ cells.

### Cleavage under targets and tagmentation assay

The CUT&Tag assay was performed using the Hyperactive Universal CUT&Tag Assay Kit (Cat#: TD904-01, Vazyme Biotech). Purified human T cells isolated from peripheral blood were first activated for 4 days with anti-CD3/CD28 Dynabeads; then, 1 × 10^5^ cells were counted and immobilized on Concanavalin A magnetic beads and permeabilized by non-ionic detergent digitonin. After successive incubation with the primary antibody (NFI-C Antibody, Cat#: 11911S, Cell Signaling Technology, 1:25) at 4 °C overnight and the secondary antibody (Goat Anti-Rabbit IgG H&L, Cat#: ab6702, Abcam, 1:100) at room temperature for 1 h, the cells were washed and incubated with protein A/G-Tn5 for 1 h. In the transposition step, adapter sequences were added to both ends of the cut fragments. Then, DNA fragments were extracted, purified, and subjected to library construction using the reagents provided by the kit. The libraries were sequenced using the Illumina NovaSeq-PE150 platform.

### Statistical analysis

Statistics were implemented using GraphPad Prism 8.0. Two-tailed unpaired/paired *t*-test and one-way analysis of variance (ANOVA) were used for comparing two and three groups, respectively. *p*-value equal to or less than 0.05 was considered statistically significant.

## Data Availability

The data presented in the study are deposited in the National genomics data center in China, accession number HRA014871.

## References

[1] Antonioli L Pacher P Vizi ES Hasko G . CD39 and CD73 in immunity and inflammation. Trends Mol Med. (2013) 19:355–67. doi: 10.1016/j.molmed.2013.03.005, PMID: 23601906 PMC3674206

[2] Borsellino G Kleinewietfeld M Di Mitri D Sternjak A Diamantini A Giometto R . Expression of ectonucleotidase CD39 by Foxp3+ Treg cells: hydrolysis of extracellular ATP and immune suppression. Blood. (2007) 110:1225–32. doi: 10.1182/blood-2006-12-064527, PMID: 17449799

[3] Cao W Fang F Gould T Li X Kim C Gustafson C . Ecto-NTPDase CD39 is a negative checkpoint that inhibits follicular helper cell generation. J Clin Invest. (2020) 130:3422–36. doi: 10.1172/JCI132417, PMID: 32452837 PMC7324201

[4] Eltzschig HK Kohler D Eckle T Kong T Robson SC Colgan SP . Central role of Sp1-regulated CD39 in hypoxia/ischemia protection. Blood. (2009) 113:224–32. doi: 10.1182/blood-2008-06-165746, PMID: 18812468 PMC2614635

[5] Fang F Cao W Mu Y Okuyama H Li L Qiu J . IL-4 prevents adenosine-mediated immunoregulation by inhibiting CD39 expression. JCI Insight. (2022) 7:157509–24. doi: 10.1172/jci.insight.157509, PMID: 35730568 PMC9309057

[6] Liao H Hyman MC Baek AE Fukase K Pinsky DJ . cAMP/CREB-mediated transcriptional regulation of ectonucleoside triphosphate diphosphohydrolase 1 (CD39) expression. J Biol Chem. (2010) 285:14791–805. doi: 10.1074/jbc.M110.116905, PMID: 20178980 PMC2863166

[7] Friedman DJ Kunzli BM YI AR Sevigny J Berberat PO Enjyoji K . From the Cover: CD39 deletion exacerbates experimental murine colitis and human polymorphisms increase susceptibility to inflammatory bowel disease. Proc Natl Acad Sci U.S.A. (2009) 106:16788–93. doi: 10.1073/pnas.0902869106, PMID: 19805374 PMC2757811

[8] Fang F Yu M Cavanagh MM Hutter Saunders J Qi Q Ye Z . Expression of CD39 on activated T cells impairs their survival in older individuals. Cell Rep. (2016) 14:1218–31. doi: 10.1016/j.celrep.2016.01.002, PMID: 26832412 PMC4851554

[9] Timperi E Barnaba V . CD39 regulation and functions in T cells. Int J Mol Sci. (2021) 22:8068–80. doi: 10.3390/ijms22158068, PMID: 34360833 PMC8348030

[10] Peres RS Liew FY Talbot J Carregaro V Oliveira RD Almeida SL . Low expression of CD39 on regulatory T cells as a biomarker for resistance to methotrexate therapy in rheumatoid arthritis. Proc Natl Acad Sci U.S.A. (2015) 112:2509–14. doi: 10.1073/pnas.1424792112, PMID: 25675517 PMC4345589

[11] Tadaka S Katsuoka F Ueki M Kojima K Makino S Saito S . 3.5KJPNv2: an allele frequency panel of 3552 Japanese individuals including the X chromosome. Hum Genome Var. (2019) 6:28. doi: 10.1038/s41439-019-0059-5, PMID: 31240104 PMC6581902

[12] Boesch M Sykora M Gasteiger S Baty F Brutsche MH Sopper S . OMIP 077: Definition of all principal human leukocyte populations using a broadly applicable 14-color panel. Cytometry A. (2022) 101:15–20. doi: 10.1002/cyto.a.24481, PMID: 34260151 PMC9292053

[13] Wilkinson ME Charenton C Nagai K . RNA splicing by the spliceosome. Annu Rev Biochem. (2020) 89:359–88. doi: 10.1146/annurev-biochem-091719-064225, PMID: 31794245

[14] Conti E Izaurralde E . Nonsense-mediated mRNA decay: molecular insights and mechanistic variations across species. Curr Opin Cell Biol. (2005) 17:316–25. doi: 10.1016/j.ceb.2005.04.005, PMID: 15901503

[15] Kervestin S Jacobson A . NMD: a multifaceted response to premature translational termination. Nat Rev Mol Cell Biol. (2012) 13:700–12. doi: 10.1038/nrm3454, PMID: 23072888 PMC3970730

[16] Caliskan M Cusanovich DA Ober C Gilad Y . The effects of EBV transformation on gene expression levels and methylation profiles. Hum Mol Genet. (2011) 20:1643–52. doi: 10.1093/hmg/ddr041, PMID: 21289059 PMC3063990

[17] Park HW Dahlin A Qiu W Tantisira KG . Gene expression changes in lymphoblastoid cell lines and primary B cells by dexamethasone. Pharmacogenet Genomics. (2019) 29:58–64. doi: 10.1097/FPC.0000000000000365, PMID: 30562215 PMC7172499

[18] Fairfax BP Makino S Radhakrishnan J Plant K Leslie S Dilthey A . Genetics of gene expression in primary immune cells identifies cell type-specific master regulators and roles of HLA alleles. Nat Genet. (2012) 44:502–10. doi: 10.1038/ng.2205, PMID: 22446964 PMC3437404

[19] Huang W Huang T Liu Y Fu J Wei X Liu D . Nuclear factor I-C disrupts cellular homeostasis between autophagy and apoptosis via miR-200b-Ambra1 in neural tube defects. Cell Death Dis. (2021) 13:17. doi: 10.1038/s41419-021-04473-2, PMID: 34930914 PMC8688449

[20] Rastogi N Gonzalez JBM Srivastava VK Alanazi B Alanazi RN Hughes OM . Nuclear factor I-C overexpression promotes monocytic development and cell survival in acute myeloid leukemia. Leukemia. (2023) 37:276–87. doi: 10.1038/s41375-022-01801-z, PMID: 36572750 PMC9898032

[21] Zhou J Wang S Qi Q Yang X Zhu E Yuan H . Nuclear factor I-C reciprocally regulates adipocyte and osteoblast differentiation via control of canonical Wnt signaling. FASEB J. (2017) 31:1939–52. doi: 10.1096/fj.201600975RR, PMID: 28122918

[22] Canale FP Ramello MC Nunez N Araujo Furlan CL Bossio SN Gorosito Serran M . CD39 expression defines cell exhaustion in tumor-infiltrating CD8(+) T cells. Cancer Res. (2018) 78:115–28. doi: 10.1158/0008-5472.CAN-16-2684, PMID: 29066514

[23] Gupta PK Godec J Wolski D Adland E Yates K Pauken KE . CD39 expression identifies terminally exhausted CD8+ T cells. PloS Pathog. (2015) 11:e1005177. doi: 10.1371/journal.ppat.1005177, PMID: 26485519 PMC4618999

[24] Simoni Y Becht E Fehlings M Loh CY Koo SL Teng KWW . Bystander CD8(+) T cells are abundant and phenotypically distinct in human tumour infiltrates. Nature. (2018) 557:575–9. doi: 10.1038/s41586-018-0130-2, PMID: 29769722

[25] Saze Z Schuler PJ Hong CS Cheng D Jackson EK Whiteside TL . Adenosine production by human B cells and B cell-mediated suppression of activated T cells. Blood. (2013) 122:9–18. doi: 10.1182/blood-2013-02-482406, PMID: 23678003 PMC3701906

[26] Figueiro F Muller L Funk S Jackson EK Battastini AM Whiteside TL . Phenotypic and functional characteristics of CD39(high) human regulatory B cells (Breg). Oncoimmunology. (2016) 5:e1082703. doi: 10.1080/2162402X.2015.1082703, PMID: 27057473 PMC4801473

[27] Qian S Xiong C Wang M Zhang Z Fu Y Hu Q . CD38(+)CD39(+) NK cells associate with HIV disease progression and negatively regulate T cell proliferation. Front Immunol. (2022) 13:946871. doi: 10.3389/fimmu.2022.946871, PMID: 36268017 PMC9577302

