# Supplementary material for: SNP rs10748643 determines CD39 expression in T and NK cells through altering NFIC binding affinity rather than interfering with RNA splicing
Source: Front Immunol. 2025 Dec 1;16:1679064. doi: 10.3389/fimmu.2025.1679064 (PMC12702745; doi:10.3389/fimmu.2025.1679064)
Supplement: Supplementary file 1 [file DataSheet1.docx]

Supplementary File

Supplemental Table 1: Primers for detecting gene expression and identifying CD39 variants

| Gene | Forward primer (5’ - 3’) | Reverse primer (5’ - 3’) |
| --- | --- | --- |
| ACTB | CATGTACGTTGCTATCCAGGC | CTCCTTAATGTCACGCACGAT |
| NFIC | ACCTGGCATACGACCTGAAC | TCCATCGAGCCCGATTTGTG |
| Variant 1/2/3/5/6/7/8/9 | ATTCTCAGGGACCCATGCTT | CCCTGGATTTCAAACTGCTG |
| Variant 4 | GCCAAGGACATTCAGCAGTTT | CCATTGAAGGCACACTGGGA |
| Variant 5 | CGAGCGGGTTTCAAGTATGG | CACGCCTGTGTCATTCTCCT |
| Variant 6 | AGAATGCAGGGTTAAAGGATGGA | TGGCACCCTGGAAGTCAAAG |
| Variant 7 | AGAAAACGTTAAGGATGGAAAGTG | CCTGGCACCCTGGAAGTCAA |
| Variant 8 | TCAGTAGGACACGACTTTCTGA | GCTTTTAGCCAAATCACCCACA |
| Variant 2 | AAGGGAACCAAGGACCTGAC | CCCACAGCAAGCAAAGCTA |
| Variant 3/9 | ATGGGGAGGGAAGAACTGT | AAGCCAAGGATGGCTAGGAT |
| CD3e | TGCTGCTGGTTTACTACTGGA | GGATGGGCTCATAGTCTGGG |
| CD19 | GGCCCGAGGAACCTCTAGT | TAAGAAGGGTTTAAGCGGGGA |

Supplemental Table 2: Antibodies for peripheral blood immune cell separation and CD39 analysis

| Antibodies | Cat. No. | Company | Clone | Reactivity | Isotype | Application | Dilution |
| --- | --- | --- | --- | --- | --- | --- | --- |
| Anti-CD3-PERCP | 317338 | Biolegend | OKT3 | Human | Mouse IgG2a, κ | FACS | 1:50 |
| Anti-CD4-Alexa Fluor 700 | 317426 | Biolegend | OKT4 | Human | Mouse IgG2b, κ | FACS | 1:50 |
| Anti-CD8-APC-cy7 | 557834 | BD | SK1 | Human | Mouse IgG1, κ | FACS | 1:50 |
| Anti-CD39-BV421 | 328214 | Biolegend | A1 | Human | Mouse IgG1, κ | FACS | 1:50 |
| Anti-CD45RO-PE | 555493 | BD | UCHL1 | Human | Mouse IgG2a, κ | FACS | 1:50 |
| Anti-CD19-BV785 | 302240 | Biolegend | HIB19 | Human | Mouse IgG1, κ | FACS | 1:50 |
| Anti-CD15-BV650 | 323034 | Biolegend | W6D3 | Human | Mouse IgG1, κ | FACS | 1:50 |
| Anti-CD56-PE-cy7 | 318318 | Biolegend | HCD56 | Human | Mouse IgG1, κ | FACS | 1:50 |
| Anti-CD14-BV711 | 563372 | BD | MφP9 | Human | Mouse IgG2b, κ | FACS | 1:50 |
| Anti-CD16-APC | 302012 | Biolegend | 3G8 | Human | Mouse IgG1, κ | FACS | 1:50 |
